# Retrospective exploratory analysis of *VEGF *polymorphisms in the prediction of benefit from first-line FOLFIRI plus bevacizumab in metastatic colorectal cancer

**DOI:** 10.1186/1471-2407-11-247

**Published:** 2011-06-14

**Authors:** Fotios Loupakis, Annamaria Ruzzo, Lisa Salvatore, Chiara Cremolini, Gianluca Masi, Paolo Frumento, Marta Schirripa, Vincenzo Catalano, Nadia Galluccio, Emanuele Canestrari, Bruno Vincenzi, Daniele Santini, Katia Bencardino, Vincenzo Ricci, Mariangela Manzoni, Marco Danova, Giuseppe Tonini, Mauro Magnani, Alfredo Falcone, Francesco Graziano

**Affiliations:** 1 U.O. Oncologia Medica 2 Universitaria, Azienda Ospedaliero-Universitaria Pisana, Istituto Toscano Tumori and Dipartimento di Oncologia, dei Trapianti e delle Nuove Tecnologie in Medicina, Università di Pisa, Italy; 2 Dipartimento di Scienze Biomolecolari, Università degli Studi "Carlo Bo", Urbino, Italy; 3 Scuola Superiore di Studi Universitari e Perfezionamento "Sant'Anna", Pisa, Italy; 4 U.O. Oncologia Medica, Ospedale di Pesaro, Italy; 5 U.O. Oncologia Medica, Università Campus Biomedico, Roma, Italy; 6 Unità di Oncologia, Istituto Scientifico San Raffaele, Milano, Italy; 7 U.O. Oncologia Medica, Fondazione IRCCS Policlinico S. Matteo, Pavia, Italy

## Abstract

**Background:**

Molecular predictors of bevacizumab efficacy in colorectal cancer have not been identified yet. Specific *VEGF *polymorphisms may affect gene transcription and therefore indirectly influence the efficacy of bevacizumab.

**Methods:**

Genomic DNA of 111 consecutive metastatic colorectal cancer patients treated with first-line FOLFIRI plus bevacizumab was obtained from blood samples. *VEGF *-2578 C/A, -1498 C/T, + 405 C/G, + 936 C/T polymorphisms were analyzed by means of PCR-RFLP. DNA samples from 107 patients treated with FOLFIRI alone served as historical control group. The relation of *VEGF *polymorphisms with PFS, evaluated through Kaplan-Meier method and log-rank test, was the primary end-point. An interaction test with a Cox model has been performed in order to demonstrate the heterogeneity of the effect of *VEGF *-1498 C/T polymorphism between bevacizumab-and control group.

**Results:**

In the bevacizumab-group median PFS and OS of patients carrying *VEGF *-1498 C/C, C/T and T/T allelic variants were, respectively, 12.8, 10.5, 7.5 months (p = 0.0046, log-rank test) and 27.3, 20.5, 18.6 months (p = 0.038, log-rank test). *VEGF *-1498 T/T genotype was associated with shorter PFS (HR = 2.13, [1.41-5.10], p = 0.0027). In the control group no significant association of *VEGF *-1498 C/T allelic variants and PFS or OS was found. Interaction between *VEGF *-1498 C/T variants and treatment effect suggested that the relation of *VEGF *-1498 T/T genotype with shorter PFS was caused by the effect of bevacizumab (p = 0.011). Other investigated polymorphisms did not affect the outcome.

**Conclusions:**

These data suggest a possible role for *VEGF *-1498 C/T variants in predicting the efficacy of bevacizumab in the up-front treatment of metastatic colorectal cancer patients. A molecular tool for selecting subjects candidate to benefit from the anti-VEGF could be important for clinical practice. The retrospective and exploratory design of the present study, coupled with the non-randomized nature of the comparison between treated and untreated patients, imply that these results should be considered as hypothesis generators. A prospective validating trial is currently ongoing.

## Background

The therapeutic approach to metastatic colorectal cancer (mCRC) patients has progressively changed in the last few years, thanks to the introduction of biologic drugs in the daily practice, such as cetuximab, a monoclonal antibody (MoAb) directed against the epidermal growth factor receptor (EGFR), and bevacizumab, a MoAb that blocks the vascular endothelial growth factor (VEGF) [1].

While it has been proven that cetuximab is not active in patients bearing *KRAS *mutant tumours [2,3], even if a recent analysis suggests that this could not be true for G13D mutations [4], up today there are no predictive biomarkers of bevacizumab efficacy. Therefore the anti-VEGF MoAb therapy is currently approved for the treatment of mCRC in association with fluoropyrimidine-based chemotherapy without any molecular selection [5].

Bevacizumab has a well-known toxicity profile causing adverse events such as bleeding, gastrointestinal perforation, arterial and venous thromboembolism, hypertension, proteinuria and wound-healing complications [6,7]. Hence, possible predictors of the efficacy of bevacizumab are needed to avoid serious adverse events at least in those patients with low chances of benefit. Up to now such determinants have not been individuated yet, despite several attempts [8-10]. Moreover, it should be considered that for *KRAS *wild-type patients the knowledge *a priori *of an intrinsic resistance to bevacizumab would lead the therapeutic choice toward the alternative option of administering the anti-EGFR cetuximab.

Many studies have demonstrated that specific *VEGF *single nucleotide polymorphisms (SNPs) may affect gene transcription with a consequent variable production of VEGF and a putative effect on pathogenesis as well as on evolution of disorders in which angiogenesis is critical [11-14]. The predictive and prognostic role of some *VEGF *SNPs has been retrospectively investigated in genomic DNA-since it has been demonstrated that the host angiogenic genotype imprints the tumor genotype [15]-of metastatic breast [16], ovarian [17], pancreatic [18] and colon cancer [19] patients treated with bevacizumab. The results regarding different polymorphisms were heterogeneous, inconclusive and inapplicable to clinical practice and often lacked of a comparison with an untreated control group. Nevertheless, it should be considered that the effect of specific genetic variants may differ among different diseases as well as on the basis of which chemotherapy is administerd together with the anti-VEGF. On the basis of such considerations, we conducted a retrospective study in order to investigate the role of four *VEGF *SNPs in predicting the efficacy of bevacizumab added to FOLFIRI as first-line treatment of mCRC patients [11,13]. The selected polymorphisms were: -2578 C/A (rs699947) and -1498 C/T (rs833061) in the promoter region; + 405 G/C (rs2010963) in the 5' untraslated region (UTR) and + 936 C/T (rs3025039) in the 3' UTR. Such allelic variants were assessed both in a population who had received FOLFIRI plus bevacizumab as first-line regimen (bevacizumab-group) and in a historical cohort of patients treated with FOLFIRI only in order to evaluate the possible interaction between *VEGF *SNPs and treatment effect.

## Methods

### Study population

Consecutive patients with histologically confirmed, metastatic colorectal adenocarcinoma receiving first-line FOLFIRI plus bevacizumab in 5 Italian Oncology Units, from December 2005 until November 2008 were included in the bevacizumab-group. The following basal characteristics were collected: sex, age (≤ or > 65 years), ECOG performance status (PS), primary tumor site (colon or rectum), surgery on primary tumor (yes or no), mucinous histology (yes or no), previous adjuvant chemotherapy (yes or no), time to metastases (synchronous or metachronous), number of involved organs (single or multiple), liver-only metastases (yes or no), baseline haemoglobin level (< or ≥ 11 g/dl), baseline leukocytes number (> or ≤ upper limit of normal, ULN), baseline lactate dehydrogenase (> or ≤ ULN), baseline alkaline phosphatase (> or ≤ ULN), baseline albumin level (< or ≥ lower limit of normal), baseline carcinoembryonic antigen (CEA, < or ≥ 100 ng/ml) and Köhne prognostic score [20].

A historical cohort of 107 patients treated from January 2001 until November 2006 with first-line FOLFIRI alone served as control group.

According to RECIST criteria version 1.0 [21], all patients were evaluated for response, progression free survival (PFS) and overall survival (OS). Bevacizumab-related toxicities were reported according to National Cancer Institute Common Terminology Criteria version 3.0 (NCI-CTCAEv3.0) [22].

Blood samples stored at -20°C were available for molecular analyses. Patients' written informed consent was required before entering the study.

The study was approved by local Ethic Committee.

### Genotyping

Genomic DNA was extracted from 1 ml of peripheral-blood samples using the "salting out" method [23]. *VEGF *-2578 C/A (rs699947), -1498 C/T (rs833061), + 405 C/G (rs2010963) and + 936 C/T (rs3025039) polymorphisms were investigated by means of polymerase chain reaction-restriction fragment lenght polymorphism (PCR-RFLP) technique (primers' sequences and restriction enzymes are specified in Table 1). Genotyping was performed by laboratory personnel blinded to patients clinical outcome.

**Table 1:** Primers and restriction enzymes used for the analysis of *VEGF *polymorphisms

Primers	Restriction enzyme	Reference SNP
*VEGF *-1498 C/T F:5'-TGT GCG TGT GGG GTT GAG ***C***G R:5'-TAC GTG CGG ACA GGG CCT GA	*Bst*UI	rs833061
*VEGF *+ 405 C/G F:5'-ATT TAT TTT TGC TTG CCA TT R:5'-GTC TGT CTG TCT GTC CGT CA	*Bsm*FI	rs2010963
*VEGF *-2578 C/A F:5'-GGC CTT AGG ACA CCA TAC C R:5'-CAC AGC TTC TCC CCT ATC C	*Bst*YI	rs699947
*VEGF *+ 936 C/T F:5'-AAG GAA GAG GAG ACT CTG CGC AGA GC R:5'-TAA ATG TAT GTA TGT GGG TGG GTG TGT CAT CAG G	*Nla*III	rs3025039

### Statistical Analysis

The primary end-point of this retrospective analysis was the relation of *VEGF *polymorphisms with PFS. The secondary end-point were the relation with OS, response rate (RR) and toxicity. PFS was defined as the time from the beginning of the treatment until the first observation of disease progression or death from any cause. Patients who underwent secondary radical surgery on metastases were censored at the time of surgery. OS was defined as the time from the beginning of the treatment until death from any cause.

All polymorphisms were examined for deviation from Hardy-Weinberg equilibrium (HWE) by comparing actual allelic distributions with those expected from HWE using a χ2-test.

A two-sided Fisher's exact test was used to evaluate the association of investigated genotypes and toxicities with RR. In both groups, Kaplan-Meier method and log-rank test were adopted to conduct an explorative analysis with the aim of estimating the importance of each clinical, pathological and genomic feature in predicting Hazard Ratios (HR) for progression and death. The most relevant covariates (those showing a p value < 0.10) were used to fit a Cox proportional hazard model. The heterogeneity of the effect of *VEGF *-1498 C/T polymorphism between bevacizumab-and control group was explored by using a statistical test for interaction, applied through a Cox model for PFS and OS. Inference on parameters of the Cox model was obtained using nonparametric bootstrap with 20000 Monte Carlo replications.

## Results

One-hundred eleven patients were included in the bevacizumab-group and received bevacizumab in combination with FOLFIRI [24] every two weeks. Patients' clinical characteristics and genotype frequencies are resumed in Table 2. All analyzed polymorphisms showed no deviation from HWE. Nine (8%), ten (9%) and 1 (1%) patients respectively developed G1, G2 and G3 hypertension during the treatment with bevacizumab. Two bowel perforations (2%), 2 arterial (2%) and 6 venous (5%) thrombotic events were observed. Out of 111 patients, 56 were partial responders and 13 were complete responders, for a global RR of 62%. Twenty-nine (26%) patients obtained a disease stabilization, with a disease control rate of 88%. At a median follow up of 13.6 months, median PFS and median OS were 10.2 and 22.2 months respectively.

**Table 2:** Association of clinical, pathological and genomic characteristics with PFS and OS in bevacizumab group

Characteristics		N (111)	Progression Free Survival			Overall Survival		
			HR	95% CI	*P*	HR	95% CI	*p*
*Sex*	Females	54	1			1		
	Males	57	0.86	0.52-1.41	0.535	1.17	0.57-2.40	0.669
*Age*	≤ 65 years	64	1			1		
	>65 years	47	1.19	0.72-1.99	0.487	1.25	0.60-2.70	0.528
*ECOG PS*	0	102	1			1		
	1-2	9	0.95	0.43-2.04	0.877	0.42	0.18-1.45	0.207
*Primary tumor*	Colon	82	1			1		
	Rectum	29	1.15	0.66-2.00	0.612	2.70	0.99-4.86	0.052
*Mucinous histology*	No	96	1			1		
	Yes	15	2.16	1.23-7.12	0.015	3.21	1.99-14.98	0.001
*Previous adjuvant CT*	No	66	1			1		
	Yes	45	1.84	1.17-3.37	0.011	1.25	0.60-2.69	0.531
*Time to metastases*	Metachronous	50	1			1		
	Synchronous	61	0.67	0.40-1.09	0.103	0.93	0.44-1.95	0.849
*No. of metastatic sites*	1	57	1			1		
	>1	54	1.22	0.75-2.02	0.421	1.77	0.85-3.62	0.130
*Liver-only metastases*	No	74	1			1		
	Yes	37	0.84	0.49-1.43	0.521	0.93	0.43-2.01	0.844
*Resected primary tumor*	No	15	1			1		
	Yes	96	2.11	0.99-3.40	0.052	1.09	0.34-3.48	0.887
*High LDH level*	No	69	1			1		
	Yes	31	0.86	0.48-1.50	0.574	1.41	0.64-3.26	0.376
	Unknown	11						
*High CEA level*	No	86	1			1		
	Yes	23	0.69	0.39-1.20	0.183	0.65	0.28-1.61	0.375
	Unknown	2						
*Low HgB level*	No	93	1			1		
	Yes	18	0.70	0.38-1.29	0.250	0.63	0.26-1.65	0.371
*Leukocytosis*	No	102	1			1		
	Yes	9	1.29	0.55-3.19	0.526	2.43	0.84-17.91	0.083
*High ALP level*	No	82	1			1		
	Yes	18	0.86	0.46-1.61	0.638	1.48	0.62-3.88	0.343
*Low Albumin level*	No	89	1			1		
	Yes	8	0.93	0.38-2.30	0.874	1.25	0.26-6.43	0.757
	Unknown	14						
*Köhne score*	Low	57	1			1		
	Intermediate-High	51	1.21	0.74-2.01	0.438	1.80	0.86-3.69	0.119
	Unknown	3						
*-2578*	*A/A*	16	1			1		
	*A/C*	60	1.02	0.50-2.07	0.959	1.19	0.41-3.38	0.753
	*C/C*	35	1.53	0.74-3.26	0.246	1.36	0.42-4.65	0.581
*-1498*	*C/C*	22	1			1		
	*C/T*	60	1.82	0.98-3.55	0.056	2.49	0.98-6.16	0.056
	*T/T*	29	2.65	1.49-6.62	0.003	2.47	0.91-7.76	0.074
*+ 405*	*G/G*	39	1			1		
	*G/C*	54	1.08	0.62-1.90	0.783	1.29	0.57-2.93	0.531
	*C/C*	18	1.11	0.52-2.39	0.771	1.36	0.47-4.12	0.544
*+ 936*	*T/T*	2	1			1		
	*C/T*	31	1.38	0.35-5.38	0.642	1.60	0.27-8.47	0.645
	*C/C*	78	1.04	0.25-4.35	0.955	1.39	0.23-7.96	0.741

Baseline characteristics of patients included in the control group are summarized in Table 3.

**Table 3:** Association of clinical, pathological and genomic characteristics with PFS and OS in the control group

Characteristics		N (107)	Progression Free Survival			Overall Survival		
			HR	95% CI	*P*	HR	95% CI	*p*
*Sex*	Females	39	1			1		
	Males	68	0.97	0.65-1.45	0.893	0.83	0.51-1.30	0.397
*Age*	≤ 65 years	60	1			1		
	>65 years	47	0.78	0.51-1.15	0.197	0.94	0.61-1.44	0.782
*ECOG PS*	0	83	1			1		
	1-2	24	1.34	0.84-2.31	0.196	2.32	1.68-6.23	0.0004
*Previous adjuvant CT*	No	75	1			1		
	Yes	32	1.13	0.74-1.75	0.562	0.77	0.49-1.22	0.268
*No. of metastatic sites*	1	57	1			1		
	>1	50	1.10	0.75-1.62	0.632	0.88	0.57-1.34	0.542
*Liver-only metastases*	No	68	1			1		
	Yes	39	0.81	0.54-1.19	0.270	0.97	0.63-1.51	0.901
*High CEA level*	No	85	1			1		
	Yes	22	1.45	0.91-2.68	0.109	1.91	1.22-4.36	0.010
*-1498*	*C/C*	25	1			1		
	*C/T*	55	1.09	0.68-1.76	0.719	0.89	0.50-1.55	0.662
	*T/T*	27	0.89	0.50-1.54	0.653	0.73	0.36-1.39	0.311

### Univariate Analysis

In the bevacizumab-group mucinous histology was significantly associated with worse PFS (HR = 2.16, 95% CI: 1.23-7.12; p = 0.015) and OS (HR = 3.21, 95% CI: 1.99-14.98; p = 0.001) (Table 2). Patients who had previously received an adjuvant chemotherapy regimen presented an increased risk for progression (HR = 1.84, 95% CI: 1.17-3.37; p = 0.011). Rectal site of primary tumor and baseline leukocytosis predicted shorter OS with a trend toward statistical significance (HR = 2.70, 95% CI: 0.99-4.86; p = 0.052 and HR = 2.43, 95% CI: 0.84-17.91; p = 0.083, respectively) (Table 2).

**Figure 1:**
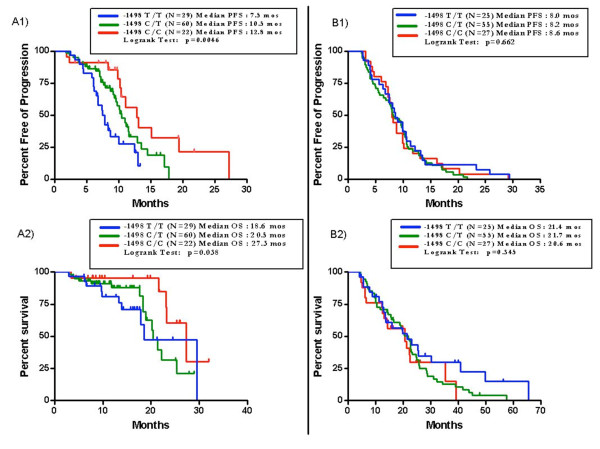
**PFS and OS according to *VEGF *-1498 C/T genotypes in the bevacizumab-group (N = 111, Panels A1 and A2) and in the control group (N = 107, Panels B1 and B2)**.

Among the analyzed *VEGF *polymorphisms, only -1498 C/T variants were significantly associated with survival. No association with RR was detected. The median PFS of patients carrying *VEGF *-1498 C/C, C/T and T/T allelic variants was 12.8, 10.5 and 7.5 months respectively (p = 0.0046, log-rank test). Median OS was 27.3, 20.5 and 18.6 months respectively (p = 0.038, log-rank test) (Figure 1-Panels A1, A2). In comparison to patients bearing *VEGF *-1498 C/C genotype those with-1498 C/T and T/T variants presented a higher risk of progression and death with an additive effect of each T allele (Table 2).

**Figure 2:**
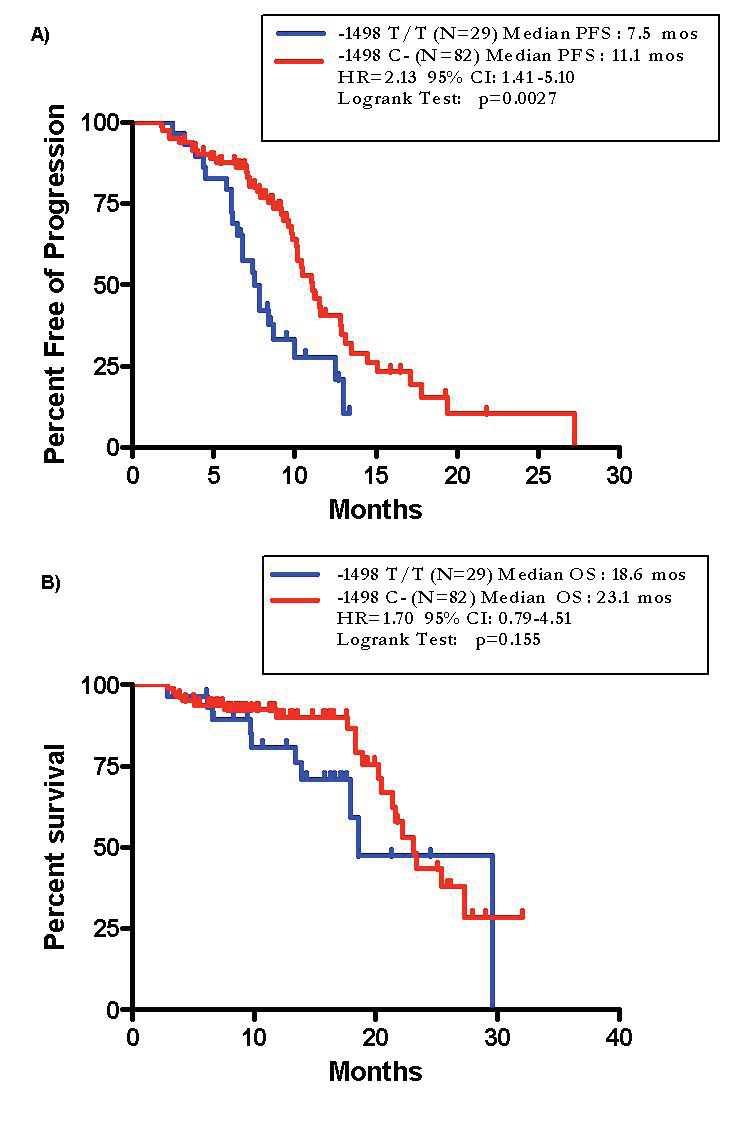
**PFS (A) and OS (B) of patients with *VEGF *-1498 T/T versus -1498 C-genotype in the bevacizumab-group (N = 111)**.

Patients bearing *VEGF *-1498 T/T genotype had significantly shorter PFS and a trend toward worse OS compared to patients carrying at least one C allele (median PFS 7.5 vs 11.1 months; HR = 2.13, 95% CI: 1.41-5.10; p = 0.0027; median OS 18.6 vs 23.1 months; HR = 1.70, 95% CI: 0.79-4.51; p = 0.155) (Figure 2).

None of *VEGF *allelic variants was significantly related to specific side-effects (i.e., hypertension, arterial or venous thrombotic events, gastrointestinal perforation).

In the control group (Table 3), among the available clinical and pathological characteristics, only ECOG PS and CEA levels were significantly related with OS. No significant correlation was found with PFS. No significant association of *VEGF *-1498 C/T polymorphism with PFS or OS was detected (PFS: p = 0.662, log-rank test; OS: p = 0.345, log-rank test) (Figure 1-Panels B1, B2)

### Cox model and Interaction Test

At the multivariate analysis, in comparison to *VEGF *-1498 C/-variants, T/T genotype retained its significant association with worse PFS (HR = 2.28, 95% CI: 1.16-4.19; p = 0.018), that translated into shorter, although not statistically significant, OS (HR = 2.22, 95% CI: 0.54-5.51; p = 0.195) (Table 4).

**Table 4:** Multivariable Cox regression model, including significant variables at the univariate analysis, and survival in bevacizumab-group

Progression Free Survival (N = 111)				
**Characteristics**		**Adjusted HR**	**95% CI**	***P***

*Mucinous histology*	No	1		
	Yes	2.45	1.19-4.44	0.016
*Previous adjuvant CT*	No	1		
	Yes	1.49	0.84-2.63	0.166
*Resected primary tumor*	No	1		
	Yes	1.59	0.51-3.71	0.383
*-1498*	*C-*	1		
	*T/T*	2.28	1.16-4.19	0.018

**Overall Survival (N = 111)**				

**Characteristics**		**Adjusted HR**	**95% CI**	**p**

*Primary tumor*	Colon	1		
	Rectum	0.40	0.15-1.60	0.245
*Mucinous histology*	No	1		
	Yes	4.22	1.60-9.27	0.009
*Leukocytosis*	No	1		
	Yes	3.02	1.03-10.07	0.043
*-1498*	*C-*	1		
	*T/T*	2.22	0.54-5.51	0.195

Also mucinous histology retained its association with shorter PFS (HR = 2.45, 95%CI: 1.19-4.44; p = 0.016) and OS (HR = 4.22, 95%CI: 1.60-9.27; p = 0.009). The interaction test between *VEGF *-1498 C/T variants and treatment effect, including both bevacizumab-group and control group, suggested that the relation of *VEGF *-1498 T/T genotype with poor outcome was significantly associated with the effect of bevacizumab in terms of PFS (p = 0.011) and with a trend toward significance with regard to OS (p = 0.081) (Table 5).

**Table 5:** Multivariable Cox regression model, including treatment, *VEGF *-1498 C/T polymorphism and their interaction as covariates

Progression Free Survival					
**Characteristics**		**N**	**Adjusted HR**	**95% CI**	***P***

*Treatment*	FOLFIRI	107	1		
	FOLFIRI + Beva	111	0.48	0.34-0.73	0.0008

***Bevacizumab-Group (N = 111)***					

*-1498*	*C-*	82	1		
	*T/T*	29	2.97	1.28-6.59	0.011

***Control Group (N = 107)***					

*-1498*	*C-*	80	1		
	*T/T*	27	0.71	0.38-1.27	0.235

**Overall Survival**					

**Characteristics**		**N**	**Adjusted HR**	**95% CI**	***P***

*Treatment*	FOLFIRI	107	1		
	FOLFIRI + Beva	111	0.50	0.29-0.94	0.033

***Bevacizumab-Group (N = 111)***					

*-1498*	*C-*	82	1		
	*T/T*	29	2.60	0.89-8.36	0.081

***Control Group (N = 107)***					

*-1498*	*C-*	80	1		
	*T/T*	27	0.66	0.31-1.31	0.239

## Discussion

Targeting VEGF is an effective strategy to inhibit the process of tumoral neoangiogenesis. Germ-line *VEGF *SNPs, in particular those affecting protein levels, could theoretically become predictors of VEGF clinical efficacy.

According to the present experience among mCRC patients treated with FOLFIRI plus bevacizumab, *VEGF *-1498 T/T allelic variant is associated with significantly shorter PFS and with a trend toward shorter OS. Such an exploratory finding at the univariate analysis has been confirmed in the multivariable Cox regression model.

On the other hand, no significant correlation of *VEGF *-1498 C/T variants with the outcome has been found in the historical control group. Although in terms of PFS the interaction test is statistically significant, thus suggesting that the impact of *VEGF *-1498 C/T SNP on the clinical outcome might be associated with the effect of bevacizumab, no definitive conclusions can be drawn from such results given the non randomized nature of the comparison. Further elusive results came from several retrospective analyses in different metastatic diseases. The retrospective study by Schneider et al. [16], performed on specimens from advanced breast cancer patients included in the phase III ECOG 2100 trial of paclitaxel plus or minus bevacizumab, found an association between *VEGF *-1154 A/G and -2578 A/C SNPs and OS among patients treated with the antiangiogenic. The interaction test confirmed the potential predictive value of *VEGF *-1154 A/G, but not the one of -2578 A/C variants [25]. However, while in ECOG 2100 trial the benefit of the addition of the anti-VEGF to weekly paclitaxel was significant in terms of RR and PFS [26], *VEGF *-1154 A/G SNP seems to significantly affect only OS, so that its role as predictor of benefit from bevacizumab is not so clear. In a phase II trial conducted in recurrent ovarian cancer patients, treated with cyclophosphamide and bevacizumab, a correlation between *VEGF *+ 936 C/T SNP and PFS has been found. In another experience, both *VEGF *and *VEGFR1 *SNPs have been analyzed in germline DNA from patients with metastatic pancreatic cancer, enrolled in a phase III randomized trial of gemcitabine and erlotinib plus or minus bevacizumab [18]. While no association of the investigated *VEGF *SNPs with clinical outcome was detected, four *VEGFR1 *SNPs were related to PFS and OS in the group treated with the anti-VEGF MoAb.

However, although our findings do not agree with the previous experiences in advanced breast, recurrent ovarian and metastatic pancreatic cancer [16-18], it should be taken into account that such results were obtained in different settings of patients affected by biologically different diseases and treated with different cytotoxic combination regimens, whose pharmacological interactions with bevacizumab and with the host angiogenic balance are not entirely clarified.

Concerning mCRC, a recent retrospective analysis performed on blood samples from 209 patients in a phase III trial comparing a first line treatment with FOLFIRI plus bevacizumab versus XELIRI plus bevacizumab, demonstrated an association of *VEGF *-2578 A/C and -1154 A/G SNPs with OS [19]. Comparing our results to the above presented study, some remarks are needed: firstly, with regard to results in terms of OS, treatments administered in second or subsequent lines influence the outcome, since a remarkable percentage of patients nowadays receive active drugs beyond the first line of treatment and, at the same time, OS was a secondary end-point of our analysis that was designed to look at PFS as primary end-point. Secondly, *VEGF *-1154 A/G was not tested in our study while *VEGF *-1498 C/T was not investigated by the hellenic group. Finally, Koutras et al. experience did not have any control group while we tested two distinct cohorts, one receiving FOLFIRI plus bevacizumab and the hystorical one treated with FOLFIRI alone, aiming at investigating the correlation of *VEGF *SNPs with the efficacy of bevacizumab treatment. It should be underlined that, as stated above, the cohorts' inception was retrospective and patients were not randomized whether receiving the anti-VEGF or not, therefore, the significance of the interaction test is affected by the non-randomization bias.

Unfortunately, we were not able to verify the effect of *VEGF *-1498 genetic variants on VEGF plasma levels and tumoral expression due to the unavailability of baseline plasma samples and tumoral tissues from our series. Since data from literature are extremely heterogeneous, it would be rather interesting to assess such a correlation in future studies.

As minor comment, in the bevacizumab-group also mucinous histology is related with shorter PFS and OS both in the univariate and in the multivariate model, thus confirming data from literature about the worse prognosis of mucinous CRCs [27,28]. Since such an association has not been verified in the historical control group due to a lack of information about it, no hypothesis may be advanced with regard to its potential predictive implication.

## Conclusions

The identification of a predictor of resistance to anti-VEGF could be crucial in the therapeutic algorythm of mCRC patients. A refined patients' selection based on molecular criteria, could avoid the exposition of patients unlikely to benefit from bevacizumab to the risk of toxicities.

At our knowledge, this is the first report to suggest a significant role for a genetic determinant in predicting bevacizumab efficacy in mCRC treated with FOLFIRI plus the anti-VEGF MoAb: patients with *VEGF *-1498 T/T genotype do not seem to benefit from antiangiogenic treatment.

Although very promising, considering the above presented limitations, these results cannot be valued as immediately applicable into clinical practice. Therefore, we consider that our data certainly deserve further investigation. To this aim, it is currently ongoing a prospective study in mCRC patients treated with first-line FOLFIRI and bevacizumab, designed with the aim of revealing a 40% risk reduction in PFS bearing *VEGF *-1498 C-variants as compared to those carrying -1498 T/T genotype. If this ongoing experience will confirm the hypothesis generated by the present retrospective series, it could be appropriate to conduct an adequately designed phase III randomized trial in order to definitively validate such preliminary considerations.

## Competing interests

The authors declare that they have no competing interests.

## Authors' contributions

FL, AR, LS, BV, DS and FG were involved in the conception and design of the study. FL, AR, LS, CC, GM, MS, VC, NG, EC, BV, DS, KB, VR, MM, MD, GT, MM, AF and FG were involved in the provision of study material and patients. FL, LS, CC, PF, MS, AF and FG did the data analysis and interpretation. FL, AF and FG were in charge of the statistical design of the study. FL, LS, CC, AR and FG wrote the manuscript. FL, AR, LS, CC, GM, PF, MS, VC, NG, EC, BV, DS, KB, VR, MM, MD, GT, MM, AF and FG approved the final version.

The authors would like to thank Dr. Elisa Romano for her kind help in revising grammar and style.

## Funding

This work was supported by Consorzio Interuniversitario per le Biotecnologie (CIB), Fanoateneo and Associazione Ricerca e Cure in Oncologia (ARCO).

## Pre-publication history

The pre-publication history for this paper can be accessed here:

http://www.biomedcentral.com/1471-2407/11/247/prepub
